# The Interaction of Large Bowel Microflora with the Colonic Mucus Barrier

**DOI:** 10.4061/2010/321426

**Published:** 2010-10-03

**Authors:** Jeffrey P. Pearson, Iain A. Brownlee

**Affiliations:** Institute for Cell and Molecular Biosciences, Medical School, Newcastle University, Newcastle upon Tyne NE2 4HH, UK

## Abstract

The colonic mucus barrier is the first line of defence that the underlying mucosa has against the wide range of potentially damaging agents of microbial, endogenous, and dietary origin that occur within the colonic lumen. The functional component of mucus is the secreted, polymeric glycoprotein mucin. The mucus barrier can either act as an energy source or a support medium for growth to the intestinal microflora. The mucus barrier appears to effectively partition the vast number of microbial cells from the underlying epithelium. The normal functionality and biochemistry of this mucus barrier appears to be lost in diseases of the colorectal mucosa. Germ-free animal studies have highlighted the necessity of the presence of the colonic microflora to drive the maturation of the colonic mucosa and normal mucus production. A number of by-products of the microflora have been suggested to be key luminal drivers of colonic mucus secretion.

## 1. Background

The colonic mucosa is constantly exposed to a wide range of luminal agents that have the potential for either mucosal damage, or mucosal protection. These luminal agents can be of microbial, dietary or endogenous origin. “Normal” colonic transit time varies widely in humans but within physiological boundaries would be between 24 and 48 hours, in comparison to transit through the upper GI tract which occurs within a few hours [1, 2]. Therefore, there is a longer exposure time of the colonic mucosa to luminal agents than to the underlying tissues of other areas of the gut. In addition, due to the role of the colon in the salvage of unabsorbed nutrients and absorption of fluid [3], these luminal agents will be concentrated (particularly in the distal bowel), resulting in further increases of mucosal exposure to these agents. While removal of water from the faecal bulk is likely to reduce the diffusion of agents from the majority of the faecal cross-section, direct contact will still occur between the mucus and the outer surfaces of the colonic luminal contents.

The large bowel also plays host to approximately 10^13^ bacteria and other micro-organisms [4] and is thought to include over 500 bacterial species [5]. As such, changes to the prevalent species within the microfloral population or to microfloral functioning and output within the bowel are likely to be intimately linked with colorectal health and disease [6]. While digestion *per se* does not tend to occur in the colon, the colonic microflora acts to degrade dietary fibre and/or other dietary factors that escape digestion to produce further agents that could either harm or protect the underlying mucosa.

The colonic mucus barrier is the first line of defence the underlying mucosa has against the myriad of damaging agents that occur within the colonic lumen [7]. The colonic mucus barrier also acts to greatly reduce the shear stress caused by the passage of the luminal bolus along the colon [8]. This barrier can also act as an energy source or as a niche for bacteria within the large bowel [9, 10]. Despite the relatively high potential for luminal exposure, previous studies in healthy humans suggest that bacteria do not routinely associate with the colonic mucosa and only occur at the luminal side of the intestinal mucus layer [11]. The remainder of this paper will focus on current evidence for how the interplay between microflora and mucus may be a key factor in mucosal health and disease and will also highlight what areas of research may be considered in the future to further understanding of this topic.

## 2. Colonic Mucus Production and Secretion

Mucus acts to protect most mucosal surfaces in the gut, airways, and urinogenital tract. The main functional component of mucus is mucin. Disulphide bridges between cysteine-rich areas towards the C- and N-termini of the mucin backbone act to endow mucus with its characteristic viscoelastic gel properties. Up to 85% of the mucin molecule is oligosaccharide side chains by weight. The terminal sugars of these side chains are believed to play a crucial role in the adhesion of mucins to different bacterial cells (e.g., [12, 13]). Changes in both the MUC gene product and glycosylation patterns are believed to be associated with the onset or development of colonic mucosal diseases, such as colorectal cancer and inflammatory bowel disease (IBD) [14].

In humans, there are five polymeric, secretory mucin gene products that are currently known, MUC2, MUC5AC, MUC5B, MUC6 [15], and MUC19 [16]. These genes for MUC2, MUC5AC, MUC5B, and MUC6 are all expressed from the same chromosome locus (11p15.5) [17]. Throughout the small and large intestine, MUC2 is the predominant mucin gene product [18]. Within the mammalian colon, mucins are highly negatively charged, due to the presence of ester sulphate and terminal sialic acids [19]. Reduction of this negative charge in secreted mucins is generally believed to be associated with colorectal disease onset and progression [20, 21].

Following transcription, mucin gene products are firstly N-glycosylated and dimerise (through cysteine-rich regions at the C-terminal of the mucin backbone) in the rough endoplasmic reticulum [22]. This N-glycosylation is also believed to be important in the subsequent transfer of mucins into the Golgi apparatus [23]. Within this compartment, mucins are O-glycosylated [24], prior to N-terminal oligomerisation [25]. Mucin granules are subsequently packaged tightly due to the presence of high levels of calcium ions [26]. Recent studies in this area have noted that the granules of the secreted gel-forming mucin MUC5B (isolated from saliva) appeal to each contain somewhere in the region of 50–100 sub-units of mucin, organised into 10–15 isolated polymers, which are believed to represent the grouping of cysteine-rich C and N-terminal regions [27]. Granules rapidly expand from a diameter of approximately 350 nm to around 1000 nm, with the end products being polymeric chains of 4–8 mucin subunits [27]. Upon their release, mucin molecules become disassociated from the calcium ions and are believed to unfurl in the presence of the aqueous milieu. It has previously been suggested that the rheologically thick mucus secretion seen in cystic fibrosis is a result of incomplete hydration of mucin granules, possibly as a result of defective HCO_3_^−^ transport [28, 29].

**Figure 1:**
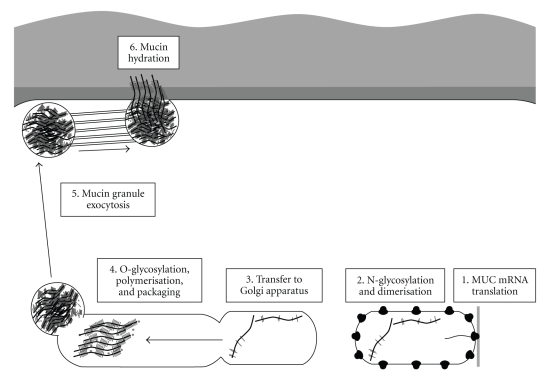
Major posttranscriptional steps involved in colonic mucin synthesis and secretion. (1) MUC gene products are translated at the rough endoplasmic reticulum. (2) MUC gene products are then N-glycosylated and dimerised at the C-terminal. (3) The N-glycosylation is necessary for mucins to be transferred to the Golgi apparatus for further processing. Within the Golgi apparatus, mucins are O-glycosylated and polymerised by disulphide bridge formation between cysteine-rich N-terminal sections of the polypeptide backbone. Polymeric mucins become tightly packed due to the presence of high concentrations of calcium ions. (4) The resulting mucin granules are externalised by the goblet cells via exocytosis. (5) Following release, mucin granules rapidly unfurl into a viscoelastic mucus gel bilayer (adapted from details given in [23, 45, 46]).

The pathways associated with mucus production are highlighted in Figure 1. Triggering of mucin synthesis or secretion, alongside goblet cell/epithelial proliferation and crypt lengthening, may be mediated by a spectrum of neurohumoral, local, and immune factors. Total mucin output from the colon can be elevated as a result of increased mucin biosynthesis, exocytosis rates, and total goblet cell numbers.

Increased MUC2 mRNA was noted by quantitative RT-PCR analysis in human colon cancer cell lines in response to a single stimulation with IL-4 (approximate two-fold increase in comparison to baseline), IL-13, and TNF-*α* (c.2.5-fold increase in MUC2 mRNA) via MAP kinase pathways [30]. N-glycosylation of MUC2 monomers appears to be necessary to drive further processing of mucin subunits, and is a required step prior to passing into the Golgi apparatus [23]. The expression of mRNA of 3 out of 8 tested isoenzymes governing O-glycosylation of mucins (polypeptide N-acetylgalactosaminyltransferases) in a colon cancer cell line, was also noted to be upregulated by the Th2 cytokine IL-4, resulting in increased incorporation of GalNAc into mucin o-glycans [31]. Within a random mutagenesis model of murine colitis, an increase in both the amounts of Th1 and Th2 cytokines secreted by cultured leukocytes and the increased leukocyte numbers within mesenteric lymph nodes were associated with the accumulation of an unglycosylated Muc2 oligomers precursor in the Golgi apparatus. Within the same study, unglycosylated MUC2 precursors were also noted to occur in human ulcerative colitis, even in noninflamed intestinal tissue [32]. In Il-10-deficient mice, total Muc2 output and synthesis was reduced in germ-free animals. Upon application of a commensal microflora, there was a significant reduction in mucin sulphation compared to sulphation in the germ-free animal [33]. This evidence would therefore suggest a major role for Th2 cytokines in the control of mucin synthesis.

In studies on isolated colonic crypts from macroscopically normal tissues, it was noted that goblet cell exocytosis, as assessed by differential interference contrast microscopy, occurred during cholinergic and histamine-mediated stimulation [34]. Within these studies, prostaglandin E_2_ stimulation did not affect mucin exocytosis from goblet cells but appeared to drive fluid loss from columnar cells, which is likely to “wash-out” mucins from the crypts to rest on the luminal surface of the epithelium. Studies on total mucin output from an isolated, vascularly perfused rat colon have noted an increase in the number of cavitated goblet cells following stimulation with cholinergic agonists, prostaglandin E_2_, and peptide YY [35]. In further studies using this model, total mucin output (as assessed by ELISA) was also increased in response to the agents used in the previous study [35] as well as serotonin, Vasoactive Intestinal Peptide (VIP), interleukin-1*β*, and NO precursors [36]. While it is not possible to isolate the effect of these factors on, for example, mucin biosynthesis or granular exocytosis within such studies, these data give a strong physiological indicator of drives for increased colonic mucin discharge.

While mostly associated with goblet cell proliferation in the lung, recent studies have suggested a role for the ETS transcription factor SPDEF in intestinal goblet cell proliferation [37, 38]. Similarly, IL-9 has been linked to the lung inflammatory pathologies. In IL-9-overexpressing mice, Muc2 expression (as well as other goblet cell-related genes) was also elevated in the intestine. Knock-out mouse studies suggested a necessity for the presence of IL-13 for this hypersecretion and goblet cell hyperplasia to occur [39].

*In vivo* studies would suggest that the colonic mucus barrier is a functional bilayer [8, 40]. The two layers are rheologically distinct [41]. Upon the application of shear stress, the outer layer of mucus rapidly moves from a gel state to a liquid state. This layer is therefore believed to act as a lubricant and is important in reducing colonic shear stress. As it is constantly removed, this outer layer may also act to return the material back into the centre of the lumen, thereby aiding in the reduction of mucosal exposure to such material [7]. The inner, adherent mucus cannot be removed by suction and is believed to act as a selective physical barrier to the contact of luminal factors with the underlying mucosa, while still allowing absorptive function to occur [42]. Due to the large hydration spheres of mucins in the hydrated mucus gel, it is likely that the mucus gel is imbued with a functional pore size anywhere in the region of 10–500 nm [43]. Diffusion through these pores will be dependent on the charge of the secreted mucins and the properties of the particle crossing the mucus, as well as the thickness of the mucus layer. Studies assessing the secretion dynamics of these mucus layers in an anaesthetised rat model would suggest that the outer, lubricative mucus layer equilibrates to maximal thicknesses of over 600 *μ*m, and the shear-resistant inner layer is maintained at approximately 100–200 *μ*m in rats fed a standard diet [8, 40, 44]. It must be noted that within these studies, the mucus layer is measured in the absence of normal colonic contents.

## 3. The Colonic Mucus Barrier as a Microbial Niche

The colonic mucus barrier can act as either an energy source, or as a potential support media for growth to the colonic microflora [47]. While a single bacterial species may not possess the necessary enzymes to cleave all the chemical linkages within the mucin structure, it has previously been hypothesised that the ability of each species to thrive as a whole may be dependent on the presence of upstream degradation of mucin by the colonic microbiome. Previous faecal culture studies under anaerobic conditions in mucin-agar gels could suggest that enterobacteria and *Bacteroides* species could be the most predominant within the colonic mucus [48]. However, it must be noted that these studies used gastric mucin as a starting point which has different carbohydrate structures than colonic mucin, which would be expected to affect both bacterial adhesion and degradation.

Due to their proximity to the underlying mucosa, it is likely that the bacteria that populate the colonic mucus barrier will have the greatest effect on colonic mucosal responses, including mucus secretion, immunity, and inflammatory responses. However, very little is known about the types of bacteria that inhabit the mucus barrier. Recently, a series of experiments outlined by Johansson et al., (2008) [49] have moved the research in this area forwards. Perhaps the major finding of this work was that within the normal mouse colonic mucus bilayer, bacteria were only found to occur within the outer, lubricative layer and did not occur within the inner adherent layer (as evidenced by 16s ribosomal RNA *in situ *hybridisation of histological sections). This would therefore suggest that under normal conditions, the adherent layer is impenetrable to colonic bacteria and that the outer layer could be a major habitat for commensal bacteria.

While colonic mucus acts as both a barrier and a potential niche for the microflora to exploit, it also appears as if the presence of bacteria in the colon is a major drive of both mucus secretion and normal colonic morphology. This is highlighted by the classic histological observation that germ-free animals have a thinner colonic musculature, with shorter colonic crypts alongside a lack of goblet cells and thin mucus layer [50–53]. In normal human development, the colonic microflora begins to develop from parturition due to indirect maternal inoculation [3]. While germ-free conditions are unlikely in either human physiology or pathophysiology, large-scale changes to bacterial numbers or content could greatly affect mucosal protection. In the case of an already deficient mucus layer, increasing numbers of bacteria that are able to degrade proteins would be more likely to cause mucosal damage/infiltration. Temporary reduction of colonic bacterial numbers (e.g., during antibiotic therapy) would be unlikely to cause unwanted mucosal side effects, but in the long term could lead to a less protective mucus barrier.

Studies in knock-out mice have suggested that deletion of the murine MUC2 orthologue Muc2 results in the onset of “spontaneous” (i.e., not chemically induced) colorectal cancer and colitis [54, 55]. Within studies on human colorectal adenoma progression, changes to mucin gene product expression and glycosylation patterns have been noted. The MUC5AC gene product, which is normally secreted in the stomach, but is absent from normal colon, is frequently found in colorectal adenomas and in the area surrounding the adenoma [20]. Mucinous and nonmucinous carcinomas exhibit separate phenotypic changes to mucin gene expression. In the mucinous adenocarcinoma, there is an increased expression of both MUC5AC and MUC2 in comparison to the nonmucinous form [56]. In the nonmucinous carcinoma, there is a reduction in total mucus output, accompanied by a shortening of the mucin oligosaccharide chains [21], particularly through the increased presence of two-residue long GalNAc -sialic acids (Sialyl-Tn antigens) [57]. The change in mucin oligosaccharides is also characterised by a reduction in sulphation levels versus normal mucosa [58] and reduced sialic acid content [59]. The loss of these factors from oligosaccharides results in the reduction of negative charge from the secreted mucin.

Similar losses in sulphation (as well as fucosylation) have also been noted to occur in ulcerative colitis [21, 60, 61]. A reduction in total MUC2 secretion also seems evident in active ulcerative colitis [62, 63]. As with colorectal cancer, there appears to be an increased expression of MUC5AC in ulcerative colitis [64, 65], which both animal and patient studies would suggest be linked to pre- or early neoplastic changes [66, 67].

The above evidence highlights that changes to both the protein and carbohydrate portions of secreted mucins occur in the diseased state. Such changes are likely to reduce the protective potential of the colonic mucus gel and may lead to an altering of the available microfloral niche within the secreted mucus, thereby potentially changing the bacterial population.

Recent preliminary metabolic profiling studies have suggested that an increased appearance of cysteine and proline occur in the faecal water extracts of individuals with colorectal cancer compared to controls [68]. Both of these amino acids are found in high amounts in mucins (cysteine is found in globular terminal structures and is necessary for polymerisation whereas proline is found in high amounts in the glycosylated variable number of tandem repeat structures, where it is thought to act as a “spacer” between glycosylated residues (serine or threonine) that imbue the molecule with a greater degree of flexibility). The increased presence of these amino acids would be suggestive of elevated mucolysis, yet would more broadly predict an increase in protein degradation in the colorectal lumen.

Certain bacterial strains appear to have the ability to preferentially target human colonic mucins [69, 70]. Adhesion to mucins is believed to be driven by the interaction between external bacterial structures and mucin carbohydrate structures. Proteomic analysis of mouse colonic mucus gels demonstrated that Fc-gamma-binding protein was found covalently bound to isolated mucins [71]. 

16s ribosomal RNA analysis has previously been used to assess global bacterial make-up of the human colonic microflora [72]. In some cases, this technology has been used to assess the occurrence of bacteria within mucosal biopsies (referred to as mucosa-associated bacteria but likely to be a mixture of any bacteria adhered to the mucosa and those associated with the outer and inner mucus layers). Such studies would suggest that the faecal microflora is distinct from that found in mucosal biopsies, with differences occurring in the mucosal biopsy microflora along the length of the large bowel [73], with marked intra-individual variations being also noted [74]. The mucosal biopsy microflora of ulcerative colitis moving from remission to relapse patients was noted to be considerably less stable over time than healthy control individuals in a small cohort study [75]. A wider diversity of the colonic biopsy TM7-bacteria was also recently noted in Crohn's Disease compared to ulcerative colitis patients or controls [76].

It is possible that there is either a direct bacterial degradation of colorectal mucins by colonic bacteria in colorectal mucosal disease or that the presence of certain bacterial species or by-products alters the pathways of mucin biosynthesis and secretion. While the relative ratios of constituent bacterial species within the colonic microflora (particularly the relative proportion of Bifidobacteria and Lactobacilli) have been postulated to be of importance to colonic health and disease [77, 78], it should be noted that the overall enzymatic spectrum [79] of the microflora or the by-products thereof [80] may be of greater relevance to human health. A number of studies have suggested that there is an increase in the numbers of mucosa-associated bacteria, as reviewed by Strober et al., (2007) [81], although these levels did not necessarily appear to correlate well with mucosal inflammation [82]. There is no consensus on large-scale changes of bacterial populations or obvious mucosal infections that occur within IBD [81] or colorectal cancer. Within the case of epithelial damage and/or infiltration, it is likely that bacterial contact with Toll-like receptors could trigger inflammatory and immune responses, including increased mucin secretion [83].

## 4. Bacterial By-Products and the Colonic Mucus Barrier

Although the colonic mucosa is surrounded by a wide variety of potentially damaging agents, the physiological response could be described as a “dampened inflammation” or general tolerance. As previously discussed, an absence of colonic microflora tends to result in a reduction in the standard maturation of the colonic epithelium, as seen in atypical histology. As very few bacteria cross the colonic epithelia (and possibly even the inner adherent mucus barrier) outside of major mucosal trauma, it is unlikely that bacterial presence is having a direct effect on colonic physiology until end-stage mucosal infection. Therefore, many of the effects of bacteria on mucus secretion may be elicited indirectly by bacterial by-products. Previous evidence from experimental models (see below) would support this hypothesis although it must be noted that only a fraction of bacterial by-products have been tested for their potential to affect such processes.

Upon bacterial cell death, lipopolysaccharides (LPS) are shed into the colonic lumen. In germ-free rats, colonic goblet cell numbers increased five days after LPS was applied orally [84]. The level of interleukin-8 (IL-8) and total mucin mRNA levels have also been shown to be significantly elevated in a mucus-producing colonic cell culture study in response to LPS stimulation [85].

**Figure 2:**
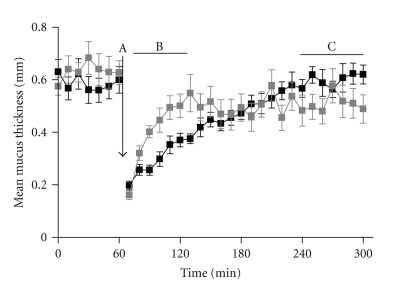
Rat colonic mucus secretion dynamics assessed in the presence of 7 mM butyrate in saline (grey line) and isotonic saline (black line). *N* = 5 animals for each treatment. After 60 minutes, the loosely adherent mucus layer is removed by suction (A). Over the subsequent 60 minutes (B), the mucus replenishment rate was approximately three times higher in the presence of butyrate versus the saline control (*P* = .0313 when compared by paired, nonparametric *t*-test). Over the last hour of assessment when the mucus barrier had reached equilibrium (C), the total maximal mucus thickness in the presence of butyrate was significantly lower than the saline control group (*P* = .0023 when compared by unpaired nonparametric *t*-test).

Both increased mucin release and increased goblet cell numbers have been reported with direct instillation of low levels of butyrate (5 mM) into a vascularly perfused rat colon model. Higher levels of butyrate (up to 100 mM) actually lowered the level of mucin secretion whereas the same concentration range of acetate (5–100 mM) increased mucin secretion in a dose-dependent manner and propionate had no effect [86]. Previous studies on mucus secretion dynamics have suggested that low luminal concentrations of butyrate (7 mM) resulted in an increased rate of mucus barrier secretion following removal, but also resulted in a decrease in the maximal mucus thickness attained (see Figure 2) [87]. Similar effects were noted in more recent studies where long-term administration of high butyrate concentrations (100 mM) directly into the mouse colon over a 7-day period resulted in an upregulation of Muc2 gene expression, but a reduction in the histologically assessed adherent mucus layer was noted [88]. Mechanistic studies have suggested that mucin output [89] and upregulation of MUC2 gene expression [90] are dependent on cholinergic pathways and myofibroblast-derived prostaglandins, respectively.

Reactive oxygen species (ROSs) are a by-product of aerobic respiration that have been shown to occur to millimolar levels in human colonic luminal contents [91]. ROS have been shown to increase mucus secretion rates at low luminal concentrations (5 mM H_2_O_2_ in the presence of Fe^++^), but lead to mucus degradation and mucosal reddening at higher concentrations (25 and 50 mM H_2_O_2_ [42]. These data would suggest that the presence of ROS could be sensed by the colonic mucosa, with the low levels driving the secretion of a more protective mucus barrier. At higher levels, the degradation of mucus, and potential damage of the underlying mucosa, results in degradation of the mucus barrier that outweighs increased secretion.

## 5. Summary

The colonic mucus bilayer acts to reduce shear stress and protect the underlying mucosa from damaging luminal entities while still allowing colonic salvage to occur. As such, the mucus barrier is a key to innate immunity. The colonic mucus barrier represents a window of colorectal health. There is evidence that the MUC gene products secreted are different in the normal state compared to colorectal pathophysiology, such as adenoma formation and ulcerative colitis.

There are uniquely high numbers of resident bacteria within the large intestine. However, the inner layer of colonic mucus appears to be generally impermeable to this resident microflora and maintains a physical barrier with an exclusion limit of 100 microns between the overlying bacteria and the underlying epithelium. The outer, lubricative mucus layer appears to act as a niche for bacterial population. 16s ribosomal RNA analysis would suggest qualitative differences between the bacterial population that resides within the mucus and that occurring within the lumen (approximated by faecal sampling). It is likely that the bacteria that reside within the mucus will have the greatest impact on the physiology and pathophysiology of the colonic mucosa.

Development of normal colonic morphology, including production of a functionally relevant mucus barrier, is largely driven by the presence of the resident colonic microflora. As bacteria rarely appear to interact directly with the colonic epithelium under normal physiological conditions, it appears likely that the diffusion of bacterial by-products across the colonic mucus barrier to the underlying mucosa acts as a major drive for mucosal maturation and hence affects the processes that govern mucus secretion (mucin synthesis, mucin granule exocytosis, and goblet cell proliferation) Previous evidence notes particular roles for LPS and SCFA in driving mucus secretion.

There is a need for further studies into how fluctuations in specific bacterial populations affect mucin synthesis and secretion, as well as how such populations adhere to or degrade mucus/mucins in a mixed culture. Coassessment of faecal mucins and bacterial populations/bacterial by-products could be utilised as a noninvasive screening technique in human participants. This methodology may be an indirect route of testing (a) whether changes in the microflora drive changes to mucin secretion/degradation and (b) whether such changes are associated with disease incidence or onset.
